# Molecular characterization of *Sarcocystis neurona* genotypes in California sea lions (*Zalophus californianus*) with polyphasic rhabdomyositis

**DOI:** 10.1371/journal.pone.0357274

**Published:** 2026-08-28

**Authors:** Alene G. Pohly, Devinn M. Sinnott, Margaret E. Martinez, Cara L. Field, Pádraig J. Duignan, Karen Shapiro

**Affiliations:** 1 Department of Pathology, Microbiology, and Immunology, School of Veterinary Medicine, University of California Davis, 1 Shields Ave, Davis, California, United States of America; 2 The Marine Mammal Center, Bunker Rd, Sausalito, California, United States of America; Guru Angad Dev Veterinary and Animal Sciences University (GADVASU), INDIA

## Abstract

A unique clinical syndrome characterized by polyphasic rhabdomyositis associated with *Sarcocystis neurona* has been reported in both free-ranging and managed care California sea lions (CSLs; *Zalophus californianus*). The pathogenesis of this disease is poorly understood. Specific *S. neurona* genotypes have been associated with more severe sarcocystosis and mortality in other marine mammals; however, *S. neurona* genotypes infecting CSLs with rhabdomyositis have not yet been investigated. The objective of this study was to characterize genotypes of *S. neurona* from CSLs with rhabdomyositis and to compare these with genotypes described in other marine and terrestrial hosts. Archived skeletal muscle samples from 25 affected free-ranging CSLs were screened for *S. neurona* DNA using polymerase chain reaction (PCR) targeting a multi-copy locus (ITS1), followed by genotype characterization using a PCR-based multilocus sequence typing approach targeting six polymorphic loci. Among these 25 CSLs, 15 different *S. neurona* genotypes were identified. Of these, six have been previously identified in southern sea otters (*Enhydra lutris nereis*) and/or other hosts, but the remaining nine have not been reported in any host to date. This study, although limited in sample size, demonstrates a wide diversity of *S. neurona* genotypes infecting free-ranging CSLs affected with rhabdomyositis, some shared with other hosts in the region, but many others that appear to be unique to sea lions.

## Introduction

The genus *Sarcocystis* represents a diverse group of apicomplexan parasites with a wide reported host range. *Sarcocystis* spp. life cycles are heteroxenous, typically involving a carnivorous definitive host that ingests the infected tissues of an herbivorous or omnivorous prey species (intermediate host) [1,2]. Sexual replication occurs within the gastrointestinal tract of definitive hosts that then shed sporocysts in the feces [1, 2]. Intermediate hosts become infected after ingesting sporocysts in fecally-contaminated food or water, with subsequent asexual replication of the parasite occurring in several tissues throughout the body, eventually forming quiescent sarcocysts encysted in skeletal muscle [1, 2].

*Sarcocystis neurona* is an important pathogen in veterinary medicine, affecting many domestic and wildlife species. The definitive host of *S. neurona* in North America is the Virginia opossum (*Didelphis virginiana*) [3,4]. Many terrestrial and aquatic species have been reported to serve as intermediate hosts including equids, nine-banded armadillos (*Dasypus novemcinctus*), skunks (*Mephitis mephitis*), sea otters (*Enhydra lutris*), and harbor porpoises (*Phocoena phocoena*) [5–7]. *Sarcocystis neurona* has been best studied as the most common etiologic agent of Equine Protozoal Myeloencephalitis (EPM), a severe neurologic disease of horses [5]. Marine mammals are believed to become infected with *S. neurona* following the land-to-sea transport of infective sporocysts from coastal watersheds inhabited by opossums into the ocean via surface water runoff [7]. *Sarcocystis neurona* has been reported as a major cause of encephalitis in marine mammals, including Pacific harbor seals (*Phoca vitulina* *richardsii*), Steller sea lions (*Eumetopias jubatus*), and southern sea otters (SSO, *Enhydra lutris nereis*), leading to stranding and death [8–11]. The intramuscular stages of *S. neurona* (sarcocysts) have been identified for decades in California sea lions (CSLs; *Zalophus californianus*) [12] as a primarily incidental finding. Since the early 2000’s, a syndrome has emerged of disseminated polyphasic rhabdomyositis that may vary in clinical expression leading to generalized muscle wasting, severe respiratory distress, or megaesophagus depending on the muscle groups affected and ultimately result in stranding and mortality from malnutrition, renal failure (myoglobinuric nephropathy) or secondary infections [13–15], TMMC unpublished data]. Histologic lesions of polyphasic rhabdomyositis include myocytes in various stages of inflammation, degeneration, necrosis, regeneration, and scarring indicative of a chronic or ongoing injury to the skeletal muscle. The pathogenesis of this condition is poorly understood, although an immune-mediated component has been proposed [14]. In SSOs and other marine mammals, specific *S. neurona* genotypes have been associated with greater disease severity and mortality (e.g., XIIIhh, IIg/j, Ib/c/d/gg) [11,16]. *Sarcocystis neurona* genotypes in CSLs with rhabdomyositis have not yet been investigated and the role of parasite genotype as a driver of this disease in this marine host is unknown.

The objectives of this study were to characterize the genotypes of *S. neurona* present in CSLs affected with polyphasic rhabdomyositis and to compare these genotypes to those reported in other marine and terrestrial mammals along the Pacific coast of North America.

## Methods

This study included CSLs that live-stranded along a 450-kilometer (280-mile) length of the central California coastline between San Francisco in the north to Morro Bay in the south, and were admitted to The Marine Mammal Center (TMMC; Sausalito, CA) for assessment. Strandings of the animals included in this study occurred through most of the year (February through October) and did not appear to be geographically clustered. Samples were only collected from animals that died in transport or while in care as a result of *Sarcocystis*- associated infection (generalized rhabdomyositis, megaesophagus, respiratory complications, myoglobinuria, protozoal cardiomyositis, meningoencephalitis, or combinations thereof) or that were euthanized under clinical veterinary oversight due to poor prognosis or moribund status based on diagnosis of the above listed morbidities. In all CSLs, *S. neurona* was identified as either the primary cause of death or a contributor to death based on gross and histologic findings. Only cases with a moderate or severe diagnosis, as previously defined [14] were included.

Medical records at TMMC were searched to generate a list of CSLs with previously confirmed clinical and histopathologic evidence of moderate to severe *S. neurona*–associated rhabdomyositis and that had archived frozen skeletal muscle samples (diaphragm, tongue, or other skeletal muscle) available for testing. Due to resource limitations, the study was limited to 25 cases. Two to four animals per year from 2017 to 2024 were randomly selected for a total of 25 cases. Postmortem examination and sample collection were performed by trained veterinary pathologists (PJD, MEM) and necropsy staff at TMMC. Diaphragm, tongue, or unspecified skeletal muscle samples from these 25 CSLs were collected at the time of necropsy by TMMC under authorized permits (NOAA CI #24359 [for the years of 2023 and 2024] and #18786−06 [for years including and prior to 2022]). The archived muscle samples were obtained as part of routine surveillance and biobanking during postmortem examination and were not collected specifically for this study. DNA was extracted in triplicate from one muscle sample (diaphragm, tongue, or unspecified skeletal muscle) for each CSL using a Qiagen DNeasy Blood and Tissue kit (Qiagen; Redwood City, CA) according to the manufacturer’s instructions with one modification: 180 µl buffer ATL and 30 µl proteinase K were added to each sample to incubate overnight at 56˚C to enhance tissue digestion. Extracted DNA samples for each CSL were screened for *S. neurona* DNA using a sensitive pan-apicomplexan nested PCR assay targeting the multi-copy ITS1 locus using previously described primers and cycling conditions [16] followed by Sanger sequencing. Forward and reverse sequences were trimmed, aligned, and compared to known reference sequences available in GenBank using BLAST (http://blast.ncbi.nlm.nih.gov). After confirming the presence of *S. neurona* DNA (>99% identity) in each CSL, one replicate of extracted DNA per animal was genotyped using a nested or hemi-nested PCR-based multilocus sequence typing (MLST) approach targeting six polymorphic loci including three surface antigens (SnSAG1/5/6, SnSAG3, SnSAG4) and three microsatellite markers (sn3, sn7, sn9). These assays were performed using previously described primers, cycling conditions, and sequence analysis methods [7,17].

The relative proportion of each genotype identified in CSLs with rhabdomyositis was calculated for the total cases included in this study (n = 25). Genotypes were then compared to previously published *S. neurona* genotypes characterized from other hosts along the Pacific coast of the United States and Canada including sea otters, harbor seals, Guadalupe fur seals (*Arctocephalus townsendi),* Steller sea lions, Northern fur seals (*Callorhinus ursinus*), Northern elephant seals (*Mirounga angustirostris*), Pacific white-sided dolphins (*Aethalodelphis obliquidens*), horses, opossums, and a harbor porpoise [7,16–19].

## Results

**Fig 1:**
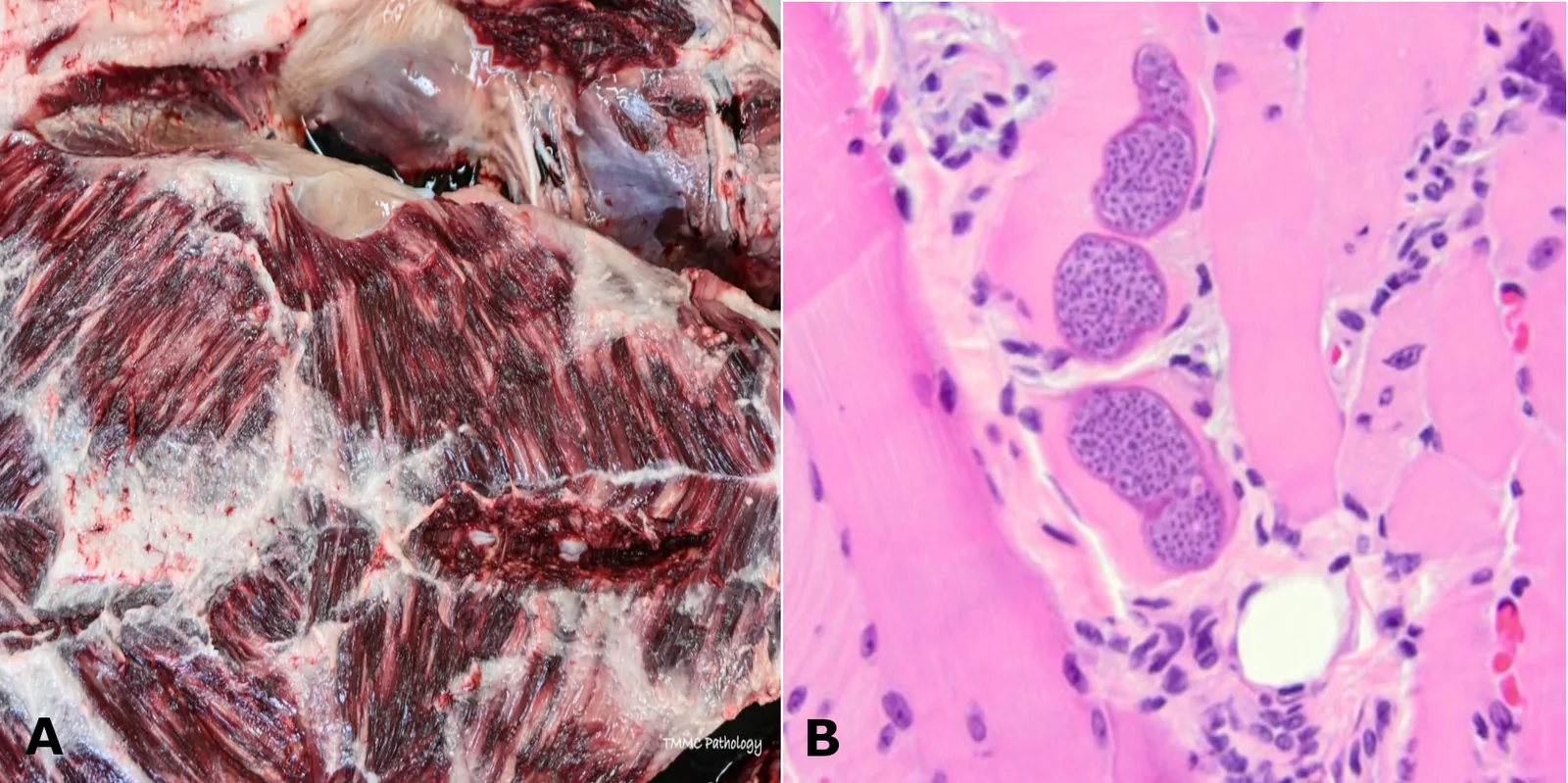
Gross and histologic features of *S. neurona*-associated rhabdomyositis in CSLs. A) California sea lion, striated muscle of the thoracic wall with pale tan linear streaking consistent with myositis. B) California sea lion striated muscle, protozoan cyst (sarcocyst) within a myocyte with loss of striation (degeneration) and surrounded by lymphocytes and plasma cells. Hematoxylin and eosin (HE).

All 25 selected cases had moderate to severe, polyphasic rhabdomyositis with intralesional sarcocysts affecting multiple skeletal muscles throughout the body, similar to what has been previously described in CSLs (Fig 1) [14]. Cases included a span of presentations from classic widely disseminated moderate to severe polyphasic rhabdomyositis to primarily megaesophagus (S1 Table). Molecular characterization across the selected six MLST loci yielded 15 different genotypes (unique combinations of antigen type and microsatellite type) (Table 1). One novel surface antigen type (XV) and five new microsatellite types (nn, oo, pp, qq, rr) were characterized that have not been previously reported in other intermediate host species [11,16–19].

**Table 1:** *Sarcocystis neurona* genotypes identified in 25 California sea lions with gross and histopathologic evidence of *S. neurona-*associated rhabdomyositis.

Animal ID	Year of death	Tissue	Surface Antigen Loci			Microsatellite Loci			Antigen type	MS type	Genotype
			SnSAG1/5/6	SnSAG3	SnSAG4	sn3	sn7	sn9			
13327	2017	muscle	1	G..T	.	12	17	18	II	g/j	IIg/j
13489	2017	muscle	6	.	G	12	17	14	VII	qq^*^	VIIqq
13305	2017	muscle	1	G..T	.	12	17	18	II	g/j	IIg/j
13909	2018	diaphragm	5	.	.	11	21	17	I	b/c/d/gg	Ib/c/d/gg
13789	2018	diaphragm	6	.	G	10	20	14	VII	w/z	VIIw/z
14282	2019	diaphragm	1	G..T	.	12	17	17	II	i	IIi
14226	2019	muscle	1	G..T	.	12	17	18	II	g/j	IIg/j
14732	2020	muscle	1	.	G	12	20	18	XV^*^	rr^*^	XVrr
14754	2020	muscle	1	G..T	.	12	17	17	II	i	IIi
14822	2020	muscle	6	.	G	14	17	14	VII	pp^*^	VIIpp
14830	2020	muscle	6	.	G	10	19	14	VII	kk	VIIkk
14987	2021	diaphragm	6	.	.	9	17	17	X	nn^*^	Xnn
14985	2021	muscle	5	.	G	11	19	14	VI	y	VIy
14953	2021	diaphragm	5	.	.	11	20	17	I	oo^*^	Ioo
15006	2021	diaphragm	5	.	.	11	21	17	I	b/c/d/gg	Ib/c/d/gg
15213	2022	muscle	5	.	.	11	22	17	I	a	Ia
15337	2022	muscle	5	.	.	11	19	14	I	y	Iy
15201	2022	diaphragm	1	G..T	.	12	17	18	II	g/j	IIg/j
15167	2022	muscle	1	G..T	.	12	17	18	II	g/j	IIg/j
15427	2023	muscle	6	.	G	11	18	14	VII	r	VIIr
15453	2023	muscle	6	.	G	11	18	14	VII	r	VIIr
15547	2023	tongue	5	.	.	11	21	17	I	b/c/d/gg	Ib/c/d/gg
15449	2023	tongue	5	G..T	G	11	18	17	III	l/m/o	III l/m/o
16065	2024	muscle	5	G..T	G	11	18	17	III	l/m/o	III l/m/o
16228	2024	muscle	1	G..T	.	12	17	18	II	g/j	IIg/j

a * indicates new surface antigen and microsatellite types identified in this study.

**Fig 2:**
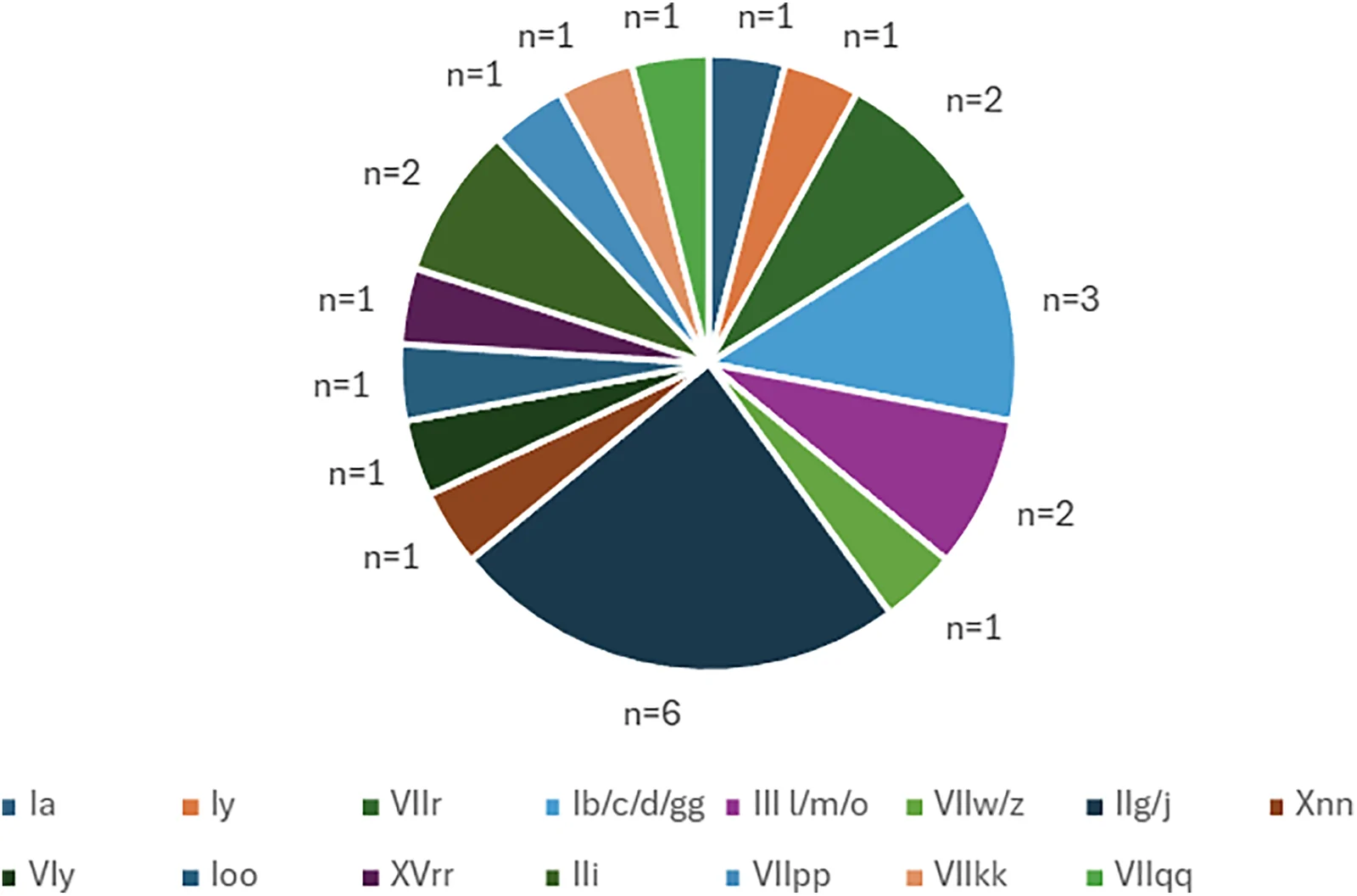
Prevalence of different *S. neurona* genotypes identified in California sea lions with *S. neurona-*associated rhabdomyositis.

The most commonly identified genotypes included IIg/j (n = 6, 24%), Ib/c/d/gg (n = 3, 12%), IIIl/m/o (n = 2, 8%), VIIr (n = 2, 8%), and IIi (n = 2, 8%) (Fig 2). Nine of the identified genotypes have not been previously reported in other marine and terrestrial species [7,17–19]. The remaining six genotypes characterized in these CSLs have been documented in SSOs, and in some cases, other marine and terrestrial mammals (Ia, Ib/c/d/gg, III l/m/o, IIg/j, VIy, IIi) [18, 19]. The Ia and Ib/c/d/gg genotypes were responsible for fatal sarcocystosis outbreaks in SSOs from Estero Bay, CA in 2004 and 2021 [16, 20]. Surface antigen type I was also associated with a clinical outbreak of meningoencephalitis in raccoons (*Procyon lotor*) in Missouri in 2014 [21]. The IIg/j genotype has been identified in SSOs from Monterey Bay, CA between 1999 and 2023, and has been linked with fatal meningoencephalitis in this species [16,18]. The IIg/j genotype has also been previously reported in several terrestrial and marine mammals in California between 1999 and 2009, including horses, opossums, harbor seals, and porpoises [11,17–19].

## Discussion

The pathogenesis, range of disease severity, and host-pathogen interactions of *S. neurona*-associated rhabdomyositis in CSLs are not fully understood. Although *S. neurona* sarcocysts can be present within skeletal muscles without causing lesions in some CSLs, prior studies have suggested a host immune-mediated etiology due to the presence of T-lymphocytes and MHC II expression in cases with severe rhabdomyositis [14]. However, the potential influence of parasite genotype on disease severity in CSLs with rhabdomyositis has not been previously explored. In other studies in marine mammals, specific genotypes of *S. neurona* and the closely related protozoal parasite *Toxoplasma gondii* have been shown to be associated with enhanced virulence, greater disease severity, or higher rates of mortality [11,16,19,22,23]. The *S. neurona* genotypes characterized in this group of CSLs with rhabdomyositis included some genotypes previously identified in other marine and terrestrial hosts, as well as nine novel genotypes that have not been reported previously.

*Sarcocystis neurona* infects marine mammals through ingestion of infective sporocysts, likely in fecally-contaminated water or prey items [7,20,24]. The definitive host, the Virginia opossum, sheds sporocysts in feces within terrestrial habitats. Sporocysts are then carried into the marine habitat through freshwater runoff [24]. In southern sea otters, increased rainfall and surface runoff has been associated with exposure to protozoa in the nearshore marine habitat and higher disease prevalence in their restricted range in central California [25]. Similar large scale epidemiologic studies investigating the effects of environmental factors on exposure to *S. neurona* in CSLs have not been performed to date. Unlike southern sea otters which are mostly found in estuarine and embayment habitats of central California that are heavily influenced by freshwater runoff, CSLs inhabit more exposed nearshore coastal waters and estuaries along the western coast of North America and often venture farther offshore into open ocean. These differences in habitat use may contribute to the differences in genotypes identified among these marine mammal hosts. Future studies characterizing *S. neurona* genotypes from opossums spanning watersheds throughout the CSL geographical range may help in identifying the terrestrial source of unique genotypes identified in CSLs and provide insight on the land-to-sea transmission dynamics and potential relationships between exposure, disease, and environmental risk factors for different clinical manifestations of sarcocystosis in CSLs.

In addition to habitat use, differences in *S. neurona* genotypes identified in this CSL population compared to southern sea otters could also be due to dietary differences between these two host species. SSOs consume filter-feeding invertebrate prey items such as shellfish and snails that have been shown to concentrate *T. gondii* oocysts, which can remain infective in shellfish for months [26,27]. Exposure to *S. neurona* has also been linked to consumption of certain clams in sea otters [28]. In contrast, the CSL diet spans a wide prey base consisting predominantly of fish and cephalopods including rockfish (*Sebastes* sp.), sardines (*Sardinops sagax*), market squid (*Loligo opalescens*), and northern anchovies (*Engraulis mordax*) [29,30]. Although these prey species do not filter feed in the same manner as bivalves, it is possible that sporocysts enter the food chain via prey species that are consumed by sea lions. Planktivorous fish, such as sardines and anchovies, have been shown, under laboratory conditions, to ingest oocysts from the water column and concentrate the pathogen in their intestinal tract, and can transmit infection to laboratory mice [31]. Given the similarity in mode of transmission for both *T. gondii* and *S. neurona* through water-borne parasite stages, it is therefore possible that these fish could be the source of infection for sea lions. Consequently, in addition to difference in coastal habitat utilization, dietary differences between CSLs and sea otters may variably affect exposure dynamics to *S. neurona*, which may thereby influence the genotypes present in these two marine predators. The risk factors that make sea otters highly susceptible to protozoal infections (including habitat and diet) are largely absent in CSLs, which should theoretically make them less susceptible to protozoal pathogens than sea otters. However, *S. neurona* continues to serve as an important source of morbidity and mortality in CSLs.

*Sarcocystis neurona* – associated encephalitis has also been reported in some pinniped species from the Pacific Northwest between 2004 and 2012, similar to that seen in southern sea otters. In these pinnipeds, including Pacific harbor seals and Steller sea lions, infections were associated predominantly with antigen types XIII and VI [11]. Only one CSL in the present study was infected with antigen type VI, and antigen type XIII was not identified in these cases. These differences in genotype distribution between CSLs in California and pinnipeds in the Pacific Northwest may reflect regional differences in sporocyst shedding by Virginia opossums or unique hydrologic features of these areas. While some genotypes overlap between CSLs and other marine mammals, only CSLs have been reported to develop severe skeletal muscle lesions while encephalitis predominates in other marine mammal hosts to date. These findings suggest a potentially unique host response to *S. neurona* in CSLs compared to other susceptible marine mammals.

Fatal infections of *S. neurona*-associated rhabdomyositis in CSLs appear to result from many different parasite genotypes. The wide diversity of genotypes identified in a relatively small sample population of 25 individuals suggests that parasite genotype alone may not be a major contributor to the development of *S. neurona*-associated rhabdomyositis and further supports the hypothesis that this syndrome in CSLs represents an immune-mediated or host-driven disease, reducing the likelihood that this disease presentation may have been associated with a novel *S. neurona* genotype. However, as noted, the sample size included in this study was small. Additionally, characterization of mixed genotype infections, if present, can be difficult to assess using MLST, as this method relies on a series of nested PCR assays that may selectively amplify DNA from a dominant genotype. Future investigations with larger sample sizes that include control groups of clinically infected and incidentally infected animals are needed to further explore the genetic diversity of *S. neurona* across the host species range, to better understand the parasite and host immune factors driving this disease, and to establish whether specific genotypes are more virulent than others in CSLs. Findings from this study may be also applied to increase the general knowledge of this pathogen for other susceptible marine and terrestrial wildlife hosts.

## Supporting information

S1 Table Clinical, demographic, and pathologic data for the 25 CSLs included in this study. (XLSX)
