# Supplementary material for: Molecular characterization of Sarcocystis neurona genotypes in California sea lions (Zalophus californianus) with polyphasic rhabdomyositis
Source: PLoS One. 2026 Aug 28;21(8):e0357274. doi: 10.1371/journal.pone.0357274 (PMC13524327; doi:10.1371/journal.pone.0357274)
Supplement: Peer review history

# Peer review: Decision Letter 0

**DOI:** 10.1371/journal.pone.0357274.r001

**Authors:** Vikrant Sudan

8 Jun 2026

Dear Dr. Shapiro,

Thank you for submitting your manuscript to PLOS ONE. After careful consideration, we feel that it has merit but does not fully meet PLOS ONE’s publication criteria as it currently stands. Therefore, we invite you to submit a revised version of the manuscript that addresses the points raised during the review process.

Please submit your revised manuscript by Jul 23 2026 11:59PM. If you will need more time than this to complete your revisions, please reply to this message or contact the journal office at plosone@plos.org. When you're ready to submit your revision, log on to https://www.editorialmanager.com/pone/ and select the 'Submissions Needing Revision' folder to locate your manuscript file.

- A letter that responds to each point raised by the academic editor and reviewer(s). You should upload this letter as a separate file labeled 'Response to Reviewers'.
- A marked-up copy of your manuscript that highlights changes made to the original version. You should upload this as a separate file labeled 'Revised Manuscript with Track Changes'.
- An unmarked version of your revised paper without tracked changes. You should upload this as a separate file labeled 'Manuscript'.

If you would like to make changes to your financial disclosure, please include your updated statement in your cover letter. Guidelines for resubmitting your figure files are available below the reviewer comments at the end of this letter.

If applicable, we recommend that you deposit your laboratory protocols in protocols.io to enhance the reproducibility of your results. Protocols.io assigns your protocol its own identifier (DOI) so that it can be cited independently in the future. For instructions see: https://journals.plos.org/plosone/s/submission-guidelines#loc-laboratory-protocols. Additionally, PLOS ONE offers an option for publishing peer-reviewed Lab Protocol articles, which describe protocols hosted on protocols.io. Read more information on sharing protocols at https://plos.org/protocols?utm_medium=editorial-email&utm_source=authorletters&utm_campaign=protocols.

As the corresponding author, your ORCID iD is verified in the submission system and will appear in the published article. PLOS supports the use of ORCID, and we encourage all coauthors to register for an ORCID iD and use it as well. Please encourage your coauthors to verify their ORCID iD within the submission system before final acceptance, as unverified ORCID iDs will not appear in the published article. *Only*  the individual author can complete the verification step; PLOS staff *cannot*  verify ORCID iDs on behalf of authors.

We look forward to receiving your revised manuscript.

Kind regards,

Vikrant Sudan, PhD

Academic Editor

PLOS One

Journal Requirements:

When submitting your revision, we need you to address these additional requirements.

1. Please ensure that your manuscript meets PLOS ONE's style requirements, including those for file naming. The PLOS ONE style templates can be found at

https://journals.plos.org/plosone/s/file?id=wjVg/PLOSOne_formatting_sample_main_body.pdf and

https://journals.plos.org/plosone/s/file?id=ba62/PLOSOne_formatting_sample_title_authors_affiliations.pdf

2. We note that your Data Availability Statement is currently as follows: “All data generated from this study is presented publicly within the submitted manuscript.”

Please confirm at this time whether or not your submission contains all raw data required to replicate the results of your study. Authors must share the “minimal data set” for their submission. PLOS defines the minimal data set to consist of the data required to replicate all study findings reported in the article, as well as related metadata and methods (https://journals.plos.org/plosone/s/data-availability#loc-minimal-data-set-definition).

For example, authors should submit the following data:

- The values behind the means, standard deviations and other measures reported;

- The values used to build graphs;

- The points extracted from images for analysis.

Authors do not need to submit their entire data set if only a portion of the data was used in the reported study.

If your submission does not contain these data, please either upload them as Supporting Information files or deposit them to a stable, public repository and provide us with the relevant URLs, DOIs, or accession numbers. For a list of recommended repositories, please see https://journals.plos.org/plosone/s/recommended-repositories.

If there are ethical or legal restrictions on sharing a de-identified data set, please explain them in detail (e.g., data contain potentially sensitive information, data are owned by a third-party organization, etc.) and who has imposed them (e.g., an ethics committee). Please also provide contact information for a data access committee, ethics committee, or other institutional body to which data requests may be sent. If data are owned by a third party, please indicate how others may request data access.

3. If the reviewer comments include a recommendation to cite specific previously published works, please review and evaluate these publications to determine whether they are relevant and should be cited. There is no requirement to cite these works unless the editor has indicated otherwise.

4. Please review your reference list to ensure that it is complete and correct. If you have cited papers that have been retracted, please include the rationale for doing so in the manuscript text, or remove these references and replace them with relevant current references. Any changes to the reference list should be mentioned in the rebuttal letter that accompanies your revised manuscript. If you need to cite a retracted article, indicate the article’s retracted status in the References list and also include a citation and full reference for the retraction notice.

Additional Editor Comments:

Please revise the manuscript as per the comments raised.

[Note: HTML markup is below. Please do not edit.]

Reviewers' comments:

Reviewer's Responses to Questions

**Comments to the Author**

1. Is the manuscript technically sound, and do the data support the conclusions?

Reviewer #1: Yes

Reviewer #2: Yes

**********

2. Has the statistical analysis been performed appropriately and rigorously?

Reviewer #1: N/A

Reviewer #2: Yes

**********

3. Have the authors made all data underlying the findings in their manuscript fully available?

The PLOS Data policy

Reviewer #1: Yes

Reviewer #2: Yes

**********

4. Is the manuscript presented in an intelligible fashion and written in standard English?

Reviewer #1: Yes

Reviewer #2: Yes

**********

Reviewer #1: Comments To Authors:

This paper is generally well written and describes the different genotypes of S. neurona identified in California sea lions with protozoal-related polyphasic myositis. The finding of many different genotypes associated with this condition is novel and publication of these results is warranted. Though this was a relatively small sample size these results serve as an important basis for developing further research projects examining the pathogenesis of this important protozoal-associated disease. The below comments and suggestions would improve clarity of the manuscript.

- Throughout suggest changing “captive” to “managed care” as the later terminology is currently more widely accepted

- Around Line 80 – It would be good to mention the specific geographic area that TMMC covers for these stranded CSLs for general reference. Also, I am assuming that the 25 CSLs that were investigated stranded throughout the range of coast that TMMC covers, however, it would be good to state that there was no geographic clustering of cases.

- Around line 83 – you could combine B and C as there isn’t much difference between these two categories of cases. Listing 3 different categories for this may lead to more confusion and is unnecessary for this paper.

- Figure Legend Line 130 – It would be good to orientate to where exactly the muscle in the gross picture is located. Is this thoracic wall?

- Figure Legend Line 131 – Can you tell for sure this is a “mature” sarcocyst given the smaller size? If not leaving out “mature” is likely fine for this paper.

- In the genotype table it would be good label the columns loci where appropriate as the abbreviations can be confusing. Similar with “MS” – microsatellite type. Can the table be formatted longitudinally for this journal so as to create more space for the headers?

- If there were two different genotypes of S. neurona present in these cases could that have been detected with the methods used? And if so and no cases had more than one genotype it would be good to state in the paper as potentially CSLs could be exposed to different genotypes of S. neurona at different time points in their lives.

- In the beginning of the Discussion around line 159 disease severity is mentioned several times, however, the specific severity of the 25 selected cases was not described. Given the large number of genotypes identified, it does not appear that genotype relates to severity of the polyphasic myositis, however, it may be good to state that the 25 selected cases all had moderate to severe polyphasic myositis if this is appropriate. This could be accomplished by stating that you only selected cases with histopathologic evidence of moderate to severe polyphasic myositis.

- Line 169 – Recommend changing to “disease being immune-mediated” Removing “more”

- Line 184 – This sentence needs to be edited or split into two sentences as the difference between site fidelity and prey is confused between otters and CSLs.

- Line 191 – Edit for grammar and punctuation

- For the Discussion section, some discussion is needed on how these results may differ from the genotype noted in some pinniped species in the Pacific NW associated with severe meningoencephalitis. And the implications of this considering the different manifestation of infection noted in these CSLs.

Reviewer #2: Dear Authors,

Your report describes the genotypes associated with a subset of S. neurona-infected California sea lions with polyphasic rhabdomyositis. You identified nine new Sn genotypes and discovered that there does not seem to be an association between genotype and presence of polyphasic rhabdomyositis. In addition, you investigated some comparisons between Sn infection risk factors in CSL and other marine intermediate hosts. Overall, I found your manuscript to be generally well-written, clear, and concise, with a few areas that I thought could use some elaboration or re-organization. Great work!

On line 32, consider changing “fifteen” to “15”.

On lines 61-63, you could consider adding information from Barbosa et al., which lists severe Sn protozoal encephalitis as a direct cause of death in a Steller sea lion as well as in several Pacific harbor seals (so, further evidence for this species), although I suppose it doesn’t specify “meningo-“ encephalitis, but rather just encephalitis.

On line 67, you first mention the “unique syndrome of severe polyphasic rhabdomyositis…”. It would be great to have a more specific definition of this term here, and especially including the significance of “polyphasic” as opposed to simply “rhabdomyositis”.

On line 69, you mention “a recent increase in severe, fatal muscular disease since 2015”, citing Seguel et al. as your reference. I think this sentence is not well-enough supported by the data, and I would recommend removing it all together. In the Seguel et al. paper, which describes polyphasic rhabdomyositis cases between 2001-2016, there is only reference to the years 2006 and 2008 having significantly higher case numbers (nothing about increases in cases during 2015 or 2016). In the Whoriskey et al., paper, it is noted that during the study period (Sept 2015-Dec 2017) the number of cases that met the inclusion criteria for the study was approximately 6 times the annual number of cases recorded during the preceding 15 years. I’m thinking this is the paper you had meant to reference there. However, I still have a little bit of a concern. First, it is not specified whether this increase is statistically significant, and second, I’m not seeing evidence that there has been a sustained increase “since 2015”, especially given that we have seen previously (in Seguel et al.), that increases can occur and then case numbers can subsequently return to baseline (i.e., I’m not seeing any data from the years 2018-present that suggest the increase is still present, and has been present “since 2015” as opposed to just fluctuating with higher case numbers in some years).

On line 101, you note that a modification was made to the manufacturer’s instructions for DNA extraction. Could you perhaps briefly specify/include why such a modification was made?

On line 121-122, you state, “All 25 selected cases had severe, polyphasic rhabdomyositis with intralesional sarcocysts affecting multiple skeletal muscles…”. Does this mean multiple skeletal muscle types across the overall sample set or multiple skeletal muscle types in each individual? If the prior (this is my guess), could I request that you reword the sentence to be a little more clear? And if the latter, please add information to the sentence starting on line 96 (“Skeletal muscle samples from these 25 CSLs…” to confirm that multiple skeletal muscle sample types from each individual were collected and analyzed.

On line 127, I think it would be pertinent to add “intermediate” between “other” and “host”, such that it reads, “…other intermediate host…”.

On line 134 (the Table 1 title), it would be ideal to have the title be more explanatory, such as “Sarcosystis neurona genotypes identified among a population of California sea lions with clinical and histopathological evidence of S. neurona-associated rhabdomyositis”, or something similar.

For your Table, it might be best to have it sideways to better fit all the title words. But perhaps it will look different in final formatting.

On line 136, for consistency, it would be great to change “types” to “genotypes”.

On lines 141-142, you note that six of the identified genotypes have been documented in southern sea otters. Later, you note additional species in which these genotypes have been documented, and I think it would be ideal to mention that here in this sentence as an overall intro, such as, “The remaining six genotypes characterized in these CSLs have been documented in southern sea otters, and in some cases, other marine and/or terrestrial species as well”, or something similar.

On lines 142-150, for each of your examples of sarcocystis in other species, could you please include the year, the general location, and whether any muscle pathology/rhabdomyositis was noted in individuals (or what the pathogenesis was)? You have some of this information for each example, but it would be great to keep it consistent, and I was definitely curious about the presence of muscle pathology in the other cases, for comparison’s sake. Additionally, I believe this entire section, beginning on line 142 at, “The Ia and Ib/c/d/gg genotypes identified in 4%...” and through the end of the paragraph, “…harbor seals, and porpoises” on line 150, belongs in the discussion section, since this content does not include your results, but rather comparison to others’ results. If this section is moved to the discussion, expanding the sentence on lines 141-142 as I recommended above, will be even more appropriate. Also, you give examples in this section for the Ia, Ib/c/d/gg, and IIg/j genotypes, and if they exist, it would be ideal to have examples for the III l/m/o, Viy, and IIi genotypes as well.

On line 146, please add the scientific name for raccoons.

On line 149, you mention “terrestrial mammals, including…”, however on line 150, you include in this list “…harbor seals, and porpoises”, which of course are not terrestrial. �

On line 151, (the Figure 2 title), it would be ideal to have it be more explanatory, such as “Sarcosystis neurona genotype frequencies in California sea lions with S. neurona-associated rhabdomyositis”, or something similar.

I think lines 166-174 (starting with, “The wide diversity…”) could be removed from here and would be better suited for the latter part of the discussion section since it’s a “future study” type statement.

In your paragraphs on lines 175-211, there is quite a bit of redundancy and also, some topics, such as diet differences between sea otters and CSLs are mentioned in two different areas. I think this whole paragraph could be tightened up a bit and reorganized such that first habitat risk factors, and then dietary risk factors are described and compared. At the end of the comparison, your sentence on line 187 starting with “The risk factors that make sea otters highly susceptible to protozoal infections…” could be a great wrap-up to that discussion; then discuss "future studies".

On line 184, I believe the “CSLs” after “California” is a typo (i.e., it should be removed).

On line 198, you reference “risk factors for fatal sarcocystosis” and I think it seems a bit out of place since your paper is focusing on polyphasic rhabdomyositis. Perhaps a simple modification to “risk factors for various forms of sarcocystis infection” or something similar.

On line 212 you also mention “fatal infections” and I was curious how many of your cases had fatal Sn-associated polyphasic rhabdomyositis? I could see how it is inferred that it was all of your cases, but I think it would be great to state that more clearly, earlier, in the results section (if the cases were all fatalities as a direct result of Sn-associated polyphasic rhabdomyositis), and how that was determined.

On line 214, I highly suggest removing “that emerged in the past decade” from the sentence (and consider removing “recently described” as well), since we know it was described at least as early as 2012 per Carlson-Bremer et al., (more than a decade ago), and the 2019 Seguel et al. paper notes that polyphasic rhabdomyositis had been a consistent but uncommon cause of stranding during the prior 15 years.

On line 216, I would change “infected” to “clinically-infected” to better differentiate these from “incidentally-infected”.

Finally, for Figure 2, I was trying to determine whether it adds useful information to your manuscript, especially since the data presented in the figure is already listed in your text, and since a black-and-white copy does not allow for color distinction. I would recommend either removing the figure all together, or keeping it but removing overlapping data from your manuscript. For example, removing the % of each genotype identified and just keeping in the “n=#”.

Overall, despite my long-winded review, I think this is a very important piece of the puzzle to have analyzed in the search for factors leading to variations in Sn presentation. Your methods were sound and analyses were straight-forward. Thank you for this contribution and I look forward to seeing your updated submission and/or publication.

**********

what does this mean?). If published, this will include your full peer review and any attached files.

If you choose “no”, your identity will remain anonymous but your review may still be made public.

**Do you want your identity to be public for this peer review?**  For information about this choice, including consent withdrawal, please see our Privacy Policy

Reviewer #1: No

Reviewer #2: No

**********

[NOTE: If reviewer comments were submitted as an attachment file, they will be attached to this email and accessible via the submission site. Please log into your account, locate the manuscript record, and check for the action link "View Attachments". If this link does not appear, there are no attachment files.]

To ensure your figures meet our technical requirements, please review our figure guidelines: https://journals.plos.org/plosone/s/figures

You may also use PLOS’s free figure tool, NAAS, to help you prepare publication quality figures: https://journals.plos.org/plosone/s/figures#loc-tools-for-figure-preparation.

NAAS will assess whether your figures meet our technical requirements by comparing each figure against our figure specifications.

# Author comment: Author response to Decision Letter 1

**DOI:** 10.1371/journal.pone.0357274.r002

31 Jul 2026

=> Please see 'response to reviewers' attachment which provides our detailed responses in a formatted approach to increase readability.

Comments from the Academic Editor:

1. Please ensure that your manuscript meets PLOS ONE's style requirements, including those for file naming.

The manuscript text and supporting figure title have been formatted according to PLOS One’s Style requirements.

2. We note that your Data Availability Statement is currently as follows: “All data generated from this study is presented publicly within the submitted manuscript.”

Please confirm at this time whether or not your submission contains all raw data required to replicate the results of your study. Authors must share the “minimal data set” for their submission. PLOS defines the minimal data set to consist of the data required to replicate all study findings reported in the article, as well as related metadata and methods (https://journals.plos.org/plosone/s/data-availability#loc-minimal-data-set-definition).

Complete genotyping results are available in Table 1. Supplemental data (S1 Table) describing the signalment, clinical history, and postmortem findings has been provided as open access to data generated from the study.

3. If the reviewer comments include a recommendation to cite specific previously published works, please review and evaluate these publications to determine whether they are relevant and should be cited. There is no requirement to cite these works unless the editor has indicated otherwise.

No additional references were recommended by the reviewers.

4. Please review your reference list to ensure that it is complete and correct. If you have cited papers that have been retracted, please include the rationale for doing so in the manuscript text, or remove these references and replace them with relevant current references. Any changes to the reference list should be mentioned in the rebuttal letter that accompanies your revised manuscript. If you need to cite a retracted article, indicate the article’s retracted status in the References list and also include a citation and full reference for the retraction notice.

References have been updated and formatted according to the style requirements.

Reviewer #1:

1. Throughout suggest changing “captive” to “managed care” as the later terminology is currently more widely accepted.

Thank you for this comment. The term “captive” has been changed to “managed care” in line 22.

2. Around Line 80 – It would be good to mention the specific geographic area that TMMC covers for these stranded CSLs for general reference. Also, I am assuming that the 25 CSLs that were investigated stranded throughout the range of coast that TMMC covers, however, it would be good to state that there was no geographic clustering of cases.

Thank you for this suggestion. The geographic range that TMMC monitors has been added to the manuscript. We have also clarified in the Methods section that the animals tested in this study did not appear to be geographically clustered.

3. Around line 83 – you could combine B and C as there isn’t much difference between these two categories of cases. Listing 3 different categories for this may lead to more confusion and is unnecessary for this paper.

We appreciate your feedback. The sentence has been adjusted to reflect that all animals included in this study were live-stranded animals that presented to The Marine Mammal Center, and samples were collected from animals that died during treatment or were euthanized under clinical veterinary supervision.

4. Figure Legend Line 130 – It would be good to orientate to where exactly the muscle in the gross picture is located. Is this thoracic wall?

Thank you for the comment. The location of the skeletal muscle in the gross picture has been added to the figure legend.

5. Figure Legend Line 131 – Can you tell for sure this is a “mature” sarcocyst given the smaller size? If not leaving out “mature” is likely fine for this paper.

We appreciate your feedback. “Mature” has been removed from the caption.

6. In the genotype table it would be good label the columns loci where appropriate as the abbreviations can be confusing. Similar with “MS” – microsatellite type. Can the table be formatted longitudinally for this journal so as to create more space for the headers?

The table headers have been edited to indicate the surface antigens and microsatellite loci, and the table has been formatted from portrait to landscape to create more space for the headers.

7. If there were two different genotypes of S. neurona present in these cases could that have been detected with the methods used? And if so and no cases had more than one genotype it would be good to state in the paper as potentially CSLs could be exposed to different genotypes of S. neurona at different time points in their lives.

We appreciate this feedback. The methods used rely on a series of nested PCR assays, which may selectively amplify DNA from a dominant genotype, precluding the characterization of mixed genotype infections. We have added a sentence to address this important limitation in the Discussion section.

8. In the beginning of the Discussion around line 159 disease severity is mentioned several times, however, the specific severity of the 25 selected cases was not described. Given the large number of genotypes identified, it does not appear that genotype relates to severity of the polyphasic myositis, however, it may be good to state that the 25 selected cases all had moderate to severe polyphasic myositis if this is appropriate. This could be accomplished by stating that you only selected cases with histopathologic evidence of moderate to severe polyphasic myositis.

Thank you for this feedback. We described selecting cases with previously confirmed clinical and histopathologic evidence of S. neurona- associated rhabdomyositis in the Methods section. We have added “moderate to severe” modifiers to the Methods section, as well as in the Results section where we discuss that the cases included a span of various clinical presentations. In response to this suggestion, we have also added a new supplemental data table that summarizes the clinical signs and postmortem findings for the 25 CSLs included in this study, including the primary contributors to death (S1 Table).

9. Line 169 – Recommend changing to “disease being immune-mediated” Removing “more”

“More” has been removed from this line.

10. Line 184 – This sentence needs to be edited or split into two sentences as the difference between site fidelity and prey is confused between otters and CSLs.

Reviewer #2 requested that these two paragraphs be edited entirely for clarity. We believe that this comment has been resolved within the context of the re-worked paragraphs.

11. Line 191 – Edit for grammar and punctuation.

Similarly, the newly edited paragraphs should address this concern.

12. For the Discussion section, some discussion is needed on how these results may differ from the genotype noted in some pinniped species in the Pacific NW associated with severe meningoencephalitis. And the implications of this considering the different manifestation of infection noted in these CSLs.

We thank you for this comment. We have added a paragraph comparing the current study’s findings to those in pinnipeds from the Pacific Northwest and the implications of these findings on our understanding of the effect of parasite genotype on rhabdomyositis in CSLs.

Reviewer #2:

1. On line 32, consider changing “fifteen” to “15”.

“Fifteen has been changed to “15” in this line.

2. On lines 61-63, you could consider adding information from Barbosa et al., which lists severe Sn protozoal encephalitis as a direct cause of death in a Steller sea lion as well as in several Pacific harbor seals (so, further evidence for this species), although I suppose it doesn’t specify “meningo-“ encephalitis, but rather just encephalitis.

Thank you for your suggestion. We have included Steller sea lions in the list of affected marine mammals and updated the citations accordingly.

3. On line 67, you first mention the “unique syndrome of severe polyphasic rhabdomyositis…”. It would be great to have a more specific definition of this term here, and especially including the significance of “polyphasic” as opposed to simply “rhabdomyositis”.

Thank you for this suggestion. Two sentences clarifying the modifier “polyphasic” and its significance have been added.

4. On line 69, you mention “a recent increase in severe, fatal muscular disease since 2015”, citing Seguel et al. as your reference. I think this sentence is not well-enough supported by the data, and I would recommend removing it all together. In the Seguel et al. paper, which describes polyphasic rhabdomyositis cases between 2001-2016, there is only reference to the years 2006 and 2008 having significantly higher case numbers (nothing about increases in cases during 2015 or 2016). In the Whoriskey et al., paper, it is noted that during the study period (Sept 2015-Dec 2017) the number of cases that met the inclusion criteria for the study was approximately 6 times the annual number of cases recorded during the preceding 15 years. I’m thinking this is the paper you had meant to reference there. However, I still have a little bit of a concern. First, it is not specified whether this increase is statistically significant, and second, I’m not seeing evidence that there has been a sustained increase “since 2015”, especially given that we have seen previously (in Seguel et al.), that increases can occur and then case numbers can subsequently return to baseline (i.e., I’m not seeing any data from the years 2018-present that suggest the increase is still present, and has been present “since 2015” as opposed to just fluctuating with higher case numbers in some years).

We understand your concern with this sentence. While we do not currently have published quantitative evidence to support the increase in cases over the last decade, there has been an observed increase in cases over this time from the authors at TMMC. The citation has been updated to include personal communication to address your concern with this statement.

5. On line 101, you note that a modification was made to the manufacturer’s instructions for DNA extraction. Could you perhaps briefly specify/include why such a modification was made?

The modification [overnight digestion in an increased volume of proteinase K] was implemented due to increased tissue digestion prior to DNA extraction. We have reported the successful application of this modification in several studies describing presence and characterization of apicomplexan protozoan pathogens in clinical tissues (Rejmanek 2010 and Sinnott 2026).

6. On line 121-122, you state, “All 25 selected cases had severe, polyphasic rhabdomyositis with intralesional sarcocysts affecting multiple skeletal muscles…”. Does this mean multiple skeletal muscle types across the overall sample set or multiple skeletal muscle types in each individual? If the prior (this is my guess), could I request that you reword the sentence to be a little more clear? And if the latter, please add information to the sentence starting on line 96 (“Skeletal muscle samples from these 25 CSLs…” to confirm that multiple skeletal muscle sample types from each individual were collected and analyzed.

Thank you for this comment. Lines 121-122 have been edited to reflect skeletal muscle at various anatomic locations within the animals. The specific sites analyzed were added to line 96.

7. On line 127, I think it would be pertinent to add “intermediate” between “other” and “host”, such that it reads, “…other intermediate host…”.

Thank you for this suggestion. “Intermediate” has been added to the sentence.

8. On line 134 (the Table 1 title), it would be ideal to have the title be more explanatory, such as “Sarcosystis neurona genotypes identified among a population of California sea lions with clinical and histopathological evidence of S. neurona-associated rhabdomyositis”, or something similar.

For your Table, it might be best to have it sideways to better fit all the title words. But perhaps it will look different in final formatting.

Thank you for the comment. The title of Table 1 has been edited to be more explanatory. The table has been re-formatted from portrait to landscape presentation to create more space for the headers.

9. On line 136, for consistency, it would be great to change “types” to “genotypes”.

We appreciate your comment. The table caption has been altered to reflect that the “types” we are referring to are the novel surface antigen and microsatellite types.

10. On lines 141-142, you note that six of the identified genotypes have been documented in southern sea otters. Later, you note additional species in which these genotypes have been documented, and I think it would be ideal to mention that here in this sentence as an overall intro, such as, “The remaining six genotypes characterized in these CSLs have been documented in southern sea otters, and in some cases, other marine and/or terrestrial species as well”, or something similar.

The sentence has been updated to include other affected species, as well.

11. On lines 142-150, for each of your examples of sarcocystis in other species, could you please include the year, the general location, and whether any muscle pathology/rhabdomyositis was noted in individuals (or what the pathogenesis was)? You have some of this information for each example, but it would be great to keep it consistent, and I was definitely curious about the presence of muscle pathology in the other cases, for comparison’s sake. Additionally, I believe this entire section, beginning on line 142 at, “The Ia and Ib/c/d/gg genotypes identified in 4%...” and through the end of the paragraph, “…harbor seals, and porpoises” on line 150, belongs in the discussion section, since this content does not include your results, but rather comparison to others’ results. If this section is moved to the discussion, expanding the sentence on lines 141-142 as I recommended above, will be even more appropriate. Also, you give examples in this section for the Ia, Ib/c/d/gg, and IIg/j genotypes, and if they exist, it would be ideal to have examples for the III l/m/o, Viy, and IIi genotypes as well.

We appreciate your thoughtful comment. One of the objectives of our study was to compare the Sarcocystis neurona genotypes identified in California sea lions to those described in other species along the Pacific coast. A brief comparison presented in the Results section demonstrates these results with respect to achieving this objective. We limit discussing this comparison in this section as we agree further interpretation should be placed in the Discussion section.

We also appreciate the suggestion to include the year, geographic location, and associated pathology for each of the listed genotypes associated with outbreaks. We have added general year ranges and geographic locations for the other marine and terrestrial host species listed. The cited references provide more details for each of these individual animals. For many of these hosts from previous studies, histopathologic findings are not described in detail. To our knowledge, CSLs are the only hosts for which severe S. neurona- associated rhabdomyositis has been described. Mild myositis has been described in sea otter infected with S. neurona, but the central nervous system appears to be the main tissue affected in clinical/fatal sarcocystosis in most hosts to date. We have added text to the Discussion acknowledging this unique clinical syndrome in CSLs compared to other hosts.

12. On line 146, please add the scientific name for raccoons.

The scientific name for racoons has been added.

13. On line 149, you mention “terrestrial mammals, including…”, however on line 150, you include in this list “…harbor seals, and porpoises”, which of course are not terrestrial. �

Thank you for your comment. The sentence has been changed to include “…terrestrial and marine mammals,…”

14. On line 151, (the Figure 2 title), it would be ideal to have it be more explanatory, such as “Sarcosystis neurona genotyp

Attachment Submitted filename: Response to Reviewers_Final.docx

# Peer review: Decision Letter 1

**DOI:** 10.1371/journal.pone.0357274.r003

**Authors:** Vikrant Sudan

16 Aug 2026

Molecular characterization of Sarcocystis neurona genotypes in California sea lions (Zalophus californianus) with polyphasic rhabdomyositis

PONE-D-26-19817R1

Dear Dr. Shapiro,

We’re pleased to inform you that your manuscript has been judged scientifically suitable for publication and will be formally accepted for publication once it meets all outstanding technical requirements.

Within one week, you’ll receive an e-mail detailing the required amendments. When these have been addressed, you’ll receive a formal acceptance letter and your manuscript will be scheduled for publication.

An invoice will be generated when your article is formally accepted. Please note, if your institution has a publishing partnership with PLOS and your article meets the relevant criteria, all or part of your publication costs will be covered. Please make sure your user information is up-to-date by logging into Editorial Manager at Editorial Manager® and clicking the ‘Update My Information' link at the top of the page. For questions related to billing, please contact billing support.

If your institution or institutions have a press office, please notify them about your upcoming paper to help maximize its impact. If they’ll be preparing press materials, please inform our press team as soon as possible -- no later than 48 hours after receiving the formal acceptance. Your manuscript will remain under strict press embargo until 2 pm Eastern Time on the date of publication. For more information, please contact onepress@plos.org.

Kind regards,

Vikrant Sudan, PhD

Academic Editor

PLOS One

Additional Editor Comments (optional):

Reviewers' comments:

Reviewer's Responses to Questions

**Comments to the Author**

Reviewer #1: All comments have been addressed

Reviewer #2: All comments have been addressed

**********

2. Is the manuscript technically sound, and do the data support the conclusions?

Reviewer #1: Yes

Reviewer #2: Yes

**********

3. Has the statistical analysis been performed appropriately and rigorously?

Reviewer #1: N/A

Reviewer #2: N/A

**********

4. Have the authors made all data underlying the findings in their manuscript fully available?

The PLOS Data policy

Reviewer #1: Yes

Reviewer #2: Yes

**********

5. Is the manuscript presented in an intelligible fashion and written in standard English?

Reviewer #1: Yes

Reviewer #2: Yes

**********

Reviewer #1: All of my comments and suggestions have been addressed. The edits to the comments strengthen the paper.

Reviewer #2: Thank you for your thorough responses to my previous questions/concerns/recommendations. Everything has been quite well addressed.

**********

what does this mean?). If published, this will include your full peer review and any attached files.

If you choose “no”, your identity will remain anonymous but your review may still be made public.

**Do you want your identity to be public for this peer review?**  For information about this choice, including consent withdrawal, please see our Privacy Policy

Reviewer #1: No

Reviewer #2: No

**********

# Editor report: Acceptance letter

**DOI:** 10.1371/journal.pone.0357274.r004

**Authors:** Vikrant Sudan

PONE-D-26-19817R1

PLOS One

Dear Dr. Shapiro,

I'm pleased to inform you that your manuscript has been deemed suitable for publication in PLOS One. Congratulations! Your manuscript is now being handed over to our production team.

At this stage, our production department will prepare your paper for publication. This includes ensuring the following:

* All references, tables, and figures are properly cited

* All relevant supporting information is included in the manuscript submission,

* There are no issues that prevent the paper from being properly typeset

You will receive further instructions from the production team, including instructions on how to review your proof when it is ready. Please keep in mind that we are working through a large volume of accepted articles, so please give us a few days to review your paper and let you know the next and final steps.

Lastly, if your institution or institutions have a press office, please let them know about your upcoming paper now to help maximize its impact. If they'll be preparing press materials, please inform our press team within the next 48 hours. Your manuscript will remain under strict press embargo until 2 pm Eastern Time on the date of publication. For more information, please contact onepress@plos.org.

You will receive an invoice from PLOS for your publication fee after your manuscript has reached the completed accept phase. If you receive an email requesting payment before acceptance or for any other service, this may be a phishing scheme. Learn how to identify phishing emails and protect your accounts at https://explore.plos.org/phishing.

If we can help with anything else, please email us at customercare@plos.org.

Thank you for submitting your work to PLOS One and supporting open access.

Kind regards,

PLOS ONE Editorial Office Staff

on behalf of

Dr. Vikrant Sudan

Academic Editor

PLOS One
